# The Stochastic Transport Dynamics of a Conserved Quantity on a Complex Network

**DOI:** 10.1038/s41598-018-32677-8

**Published:** 2018-09-24

**Authors:** Pablo Medina, Jaime Clark, Miguel Kiwi, Felipe Torres, José Rogan, Juan Alejandro Valdivia

**Affiliations:** 1 Departamento de Física, Facultad de Ciencias, Universidad de Chile, Santiago de Chile, Chile; 2 Facultad de Física, Pontificia Universidad Católica de Chile, Santiago de Chile, Chile; 3 Centro para el Desarrollo de la Nanociencia y Nanotecnología, Santiago, Chile

## Abstract

The stochastic dynamics of conserved quantities is an emergent phenomena observed in many complex systems, ranging from social and to biological networks. Using an extension of the Ehrenfest urn model on a complex network, over which a conserved quantity is transported in a random fashion, we study the dynamics of many elementary packets transported through the network by means of a master equation approach and compare with the mean field approximation and stochastic simulations. By use of the mean field theory, it is possible to compute an approximation to the ensemble average evolution of the number of packets in each node which, in the thermodynamic limit, agrees quite well with the results of the master equation. However, the master equation gives a more complete description of the stochastic system and provides a probabilistic view of the occupation number at each node. Of particular relevance is the standard deviation of the occupation number at each node, which is not uniform for a complex network. We analyze and compare different network topologies (*small world, scale free, Erdos*-*Renyi*, among others). Given the computational complexity of directly evaluating the asymptotic, or equilibrium, occupation number probability distribution, we propose a scaling relation with the number of packets in the network, that allows to construct the asymptotic probability distributions from the network with one packet. The approximation, which relies on the same matrix found in the mean field approach, becomes increasingly more accurate for a large number of packets.

## Introduction

One of the most interesting and fundamental problem in physics is related to the understanding of how the reversible microphysics gives rise to irreversible thermodynamics. An important model that has contributed to this comprehension is the “Ehrenfest urn”^1^, that was proposed in 1907 and solved exactly^2,3^ in 1947. In this model, we have N marbles, or packets, that move randomly and in a conserved manner between two urns, so that at each time step a packet is selected at random and changed from the original to the other urn.

Here we elaborate on a generalization of the Ehrenfest model by Clark *et al*.^4^, in which a number of urns are interconnected as a complex network, and the marbles or packets can only jump to urns to which the first one has a directed connection in a conservative manner.

In this respect, there has been a large amount of published research about complex networks in areas as diverse as physics, biology, and social sciences^5–8^. Originally, the interest was on the topology of the networks, such as their characterization in terms of their connectivity distribution *P*(*k*). For example, the study of *scale-free networks*, which follow a power law $P(k)\sim {k}^{-\alpha }$ for large values of *k*, became very popular^9–18^. Models based on preferential attachment seem to explain a diversity of power law exponents^5,19–21^. Another characterization of such networks involves the distribution of the shortest distance between nodes, and a noteworthy example are *small-world networks*^22^ that have short average distances. More recently, researches have began to study the topological evolution of these networks^7,8^, or as dynamical systems over which a certain quantity is transported^9,23–37^.

The generalization of the “Ehrenfest urn” to a complex network, as proposed by Clark *et al*.^4^, is an example of the nontrivial transport that can occur on a complex network^38–41^, and should have similar properties to traffic in cities, electric networks, etc.

In this manuscript we construct the master equation that describes the evolution of the occupation number probability at each urn, and then compare the results with a stochastic simulation of the network of urns and the mean field approach proposed by^4^. The mean field theory approximation to the ensemble average evolution of the number of packets in each node agrees quite well with the results of the master equation, particularly in the thermodynamic limit. However, the master equation gives a more complete description of the stochastic system, and provides a probabilistic view of the occupation number on each node. Of particular relevance is the standard deviation of the occupation number at each node, which is not uniform for a complex network, and therefore provides an intriguing result from a statistical mechanics point of view.

Given the computational complexity of directly evaluating the asymptotic, or equilibrium, occupation number probability distribution; we propose a scaling relation with the number of packets in the network that allows to construct the asymptotic probability distributions from the network with one packet. Interestingly enough, the scaling approach requires the same matrix that is constructed for the mean field approach. We will notice that the approximation becomes increasingly more accurate as the number of packets becomes large.

## Results

### The model

The “Ehrenfest urn” over a complex network, as generalized by Clark *et al*.^4^, describes the transport of *N* packets between the *M* nodes of a directed network. At a given time *t* a random packet, which is at a node *i*, is chosen to move to one of the *k*_*i*_ nodes to which node *i* is connected, i.e., in its *outgoing set*. Similarly, the *incoming set* of node *i* corresponds to the nodes that connect to node *i*. Hence, the dynamics of the packets is conserved, so that at a given time we have *x*_*i*_(*t*) packets at the *i*^*th*^ node, with the restriction

1 $$N=\sum _{i=1}^{M}\,{x}_{i}(t\mathrm{).}$$

**Figure 1:**
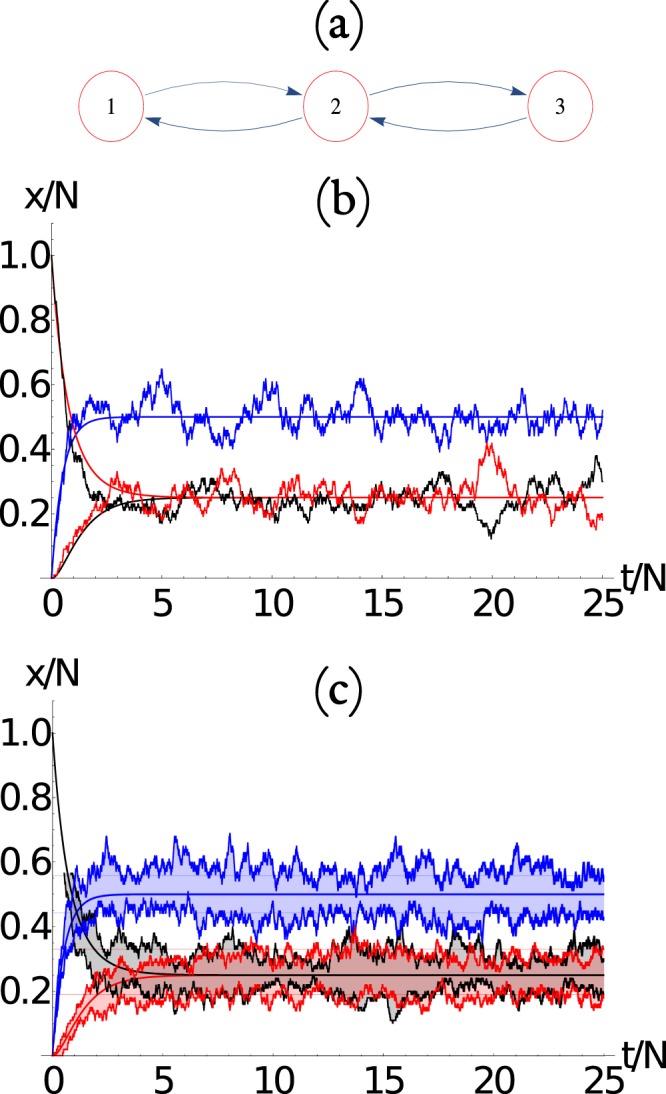
(**a**) Three-node network, in which the arrows determine the directed connectivity of the network. (**b**) Time evolution of the number of packets at each node for a stochastic simulation using *N* = 100. We show the evolution for node 1 (red), 2 (blue), and 3 (black). The initial condition is *m*_1_(0) = *N*, *m*_2_(0) = *m*_3_(0) = 0. (**c**) The time evolution of the number of packets at each node for 10 stochastic simulations (similar to b), showing that the system evolves to an asymptotic state that presents fluctuations around a mean value (thick color lines) that can be obtained from the *λ*_0_ = 0 eigenvector of the *B* matrix as described in the text. The standard deviation around the mean (thin color lines) for each node is constructed analytically from the master equation as described in the text. The thick color lines correspond to the maximum and minimum value of the 10 simulations for each node at each time.

In Fig. 1(a) we show a 3-node network that is represented by the adjacency matrix *A*

2 $$A=[\begin{array}{rrr}0 & 1 & 0\\ 1 & 0 & 1\\ 0 & 1 & 0\end{array}]\mathrm{.}$$

Here *A*_*i*,*j*_ = 1 if there is a directed connection from node *i* to node *j*, and 0 otherwise. The size of the *outgoing set* of the *i*^*th*^ node is then ${k}_{i}={\sum }_{j}\,{A}_{i,j}$. A stochastic simulation of the packet transport for *N* = 100 is plotted in Fig. 1(b), which shows that the system relaxes to an asymptotic state that is not uniform, e.g., the middle node has on average twice as many packets than the other 2 nodes. This results may have implications in many fields such as traffic in cities, lines in supermarkets, etc. Of course, we also see fluctuations around the mean asymptotic solution. This is much clearer in Fig. 1c where we display 10 simulations for the same network. Below, we find a way to describe these fluctuations, and in fact the whole probability distribution, with the help of a master equation.

Following the mean field approach proposed by Clark *et al*.^4^ one assumes that evaluating an ensemble average evolution 〈*m*_*i*_(*t*)〉 of *x*_*i*_(*t*), in the thermodynamic limit, is equivalent to assume that all the *N* packets move to a new node in a time *N*, so that the evolution equation for the ensemble average is

3 $$\langle {m}_{i}(t+\mathrm{1)}\rangle -\langle {m}_{i}(t)\rangle \approx \frac{1}{N}(-\langle {m}_{i}(t)\rangle +\sum _{j\ne i}^{N}\,\frac{{A}_{ji}}{{k}_{j}}\langle {m}_{j}(t)\rangle ).$$

The first term on the right represents the transport of the 〈*m*_*i*_〉 packets to the *outgoing set* of the *i*^*th*^ node. The second term represents the packets that get transported to the *i*^*th*^ node from all the nodes that have the *i*^*th*^ node in their *outgoing sets*. Of course, this is properly normalized by the size of each of the *outgoing sets*.

For large *N* we can approximate this expression by a time derivative, so that we can write it in vectorial form as

4 $$\frac{d}{dt}\langle \overrightarrow{{\bf{m}}}(t)\rangle =\frac{1}{N}{\rm{B}}\,\langle \overrightarrow{{\bf{m}}}(t)\rangle ,$$

where 〈$\overrightarrow{m}$(*t*)〉 → {〈*m*_1_(*t*)〉, 〈*m*_2_(*t*)〉, ..., 〈*m*_*M*_(*t*)〉}, and B is the dynamical matrix whose elements are

5 $${B}_{i,j}=-\,{\delta }_{i,j}+\frac{{A}_{j,i}}{{k}_{j}},$$

where *δ*_*i*,*j*_ is the Kronecker delta. Hence, given an initial condition, we can evaluate 〈$\overrightarrow{{\rm{m}}}$(*t*)〉 by integrating the above equation, to obtain

6 $$\langle \overrightarrow{{\bf{m}}}(t)\rangle ={e}^{{\bf{B}}t/N}\,\langle \overrightarrow{{\bf{m}}}\mathrm{(0)}\rangle $$

which can be obtained by diagonalizing the dynamical matrix **B**, such that

7 $${\bf{B}}={{\bf{V}}}^{-1}{\boldsymbol{\Lambda }}{\bf{V}},$$

and writing

8 $$\langle \overrightarrow{{\bf{m}}}(t)\rangle ={{\bf{V}}}^{-1}\,{e}^{{\rm{\Lambda }}t/N}\,{\bf{V}}\,\langle \overrightarrow{{\bf{m}}}(t)\rangle ,$$

where Λ is the diagonal matrix of the eigenvalues {*λ*_1_, *λ*_2_, ..., *λ*_*M*_} of **B**, and **V** is the matrix of column eigenvectors. Therefore, the time evolution of the particle number is dominated by the smallest nonzero eigenvalue $\tilde{<mml:mpadded xmlns:xlink="http://www.w3.org/1999/xlink" voffset="0">\lambda</mml:mpadded>}$, namely

9 $$\langle {m}_{i}(t)\rangle ={e}^{-|\Re [\tilde{<mml:mpadded xmlns:xlink="http://www.w3.org/1999/xlink" voffset="0">\lambda</mml:mpadded>}]|t/N}\langle {m}_{i}\mathrm{(0)}\rangle ,$$

where $|\Re [\tilde{<mml:mpadded xmlns:xlink="http://www.w3.org/1999/xlink" voffset="0">\lambda</mml:mpadded>}]|$ is the absolute value of the real part of the $\tilde{<mml:mpadded xmlns:xlink="http://www.w3.org/1999/xlink" voffset="0">\lambda</mml:mpadded>}$. The eigenvalues of the matrix **B** give the timescales of the system, as studied in detail in ref.^4^ for different types of complex networks. Also from the *λ* = 0 eigenvector (*t* → ∞), we can evaluate the asymptotic ensemble average occupation number. Such eigenstate exists because

$$\sum _{i=1}^{M}\,\langle {m}_{i}(t)\rangle =N\mathrm{.}$$

The eigenvalues for matrix **B** of the network of Fig. 1(a) are *λ*_1_ = −2, *λ*_2_ = −1, and *λ*_0_ = 0, hence the relaxation time of the system is *τ* = *N*. The evolution of the dynamics is shown as the continuous lines of Fig. 1(b). The asymptotic state can be recovered from the *λ*_0_ = 0 eigenvector $\tilde{v}$ = {1, 2, 1}/5, so that the asymptotic state is 〈*m*_1_〉 = 〈*m*_3_〉 = *N*/4 and 〈*m*_2_〉 = *N*/2. The existence of such nonuniform asymptotic states is an interesting results in view of what we know about equilibrium statistical mechanics.

It is interesting to note that the *M* = 2 solution corresponds to the original “Ehrenfest urn” solution constructed by Marc Kac^2,3^. The mean field approximation improves as we increase *N*, however we cannot evaluate the fluctuations within this approximation, and we need to resort to a master equation for the probability of occupation.

Let us notice that the mean field evolution equation can be cast into a rate equation for the particle number variation, given by

10 $$\frac{d}{dt}\langle {m}_{i}(t)\rangle =\sum _{j}\,({p}_{j,i}\langle {m}_{j}(t)\rangle -{p}_{i,j}\langle {m}_{i}(t)\rangle ),$$

where *p*_*i*,*j*_ is the transition probability of one packet from the *i*−*th* node to the *j*−*th* node. Notice that the conservation of the particle number can be obtained directly from Eq. (10); indeed

11 $$\frac{d}{dt}\sum _{i}\,\langle {m}_{i}(t)\rangle =\sum _{i,j}\,({p}_{j,i}\langle {m}_{j}(t)\rangle -{p}_{i,j}\langle {m}_{i}(t))=0.$$

Replacing *p*_*i*,*j*_ = *A*_*i*,*j*_/(*Nk*_*i*_) into the Eq. (10), we obtain

12 $$\begin{array}{c}\frac{d}{dt}\langle {m}_{i}(t)\rangle =\frac{1}{N}(\sum _{j}\,\frac{{A}_{j,i}}{{k}_{j}}\langle {m}_{j}(t)\rangle -\sum _{j}\,\frac{{A}_{i,j}}{{k}_{i}}\langle {m}_{i}(t)\rangle ),\\ \,\,\,\,=\frac{1}{N}(\sum _{j}\,\frac{{A}_{j,i}}{{k}_{j}}\langle {m}_{j}(t)\rangle -\langle {m}_{i}(t)\rangle )\end{array}$$

which is equivalent to the mean field equation, since ${\sum }_{j}\,{A}_{i,j}={k}_{i}$ in the last term.

Close to the steady state condition, Eq. (12) can be written as

13 $$\langle {m}_{i}(\tau )\rangle =\sum _{j}\,\frac{{A}_{j,i}}{{k}_{j}}\langle {m}_{j}(\tau )\rangle .$$

Therefore, for an undirected network (*outgoing set* is equal to *incoming set* of each node), a solution can be written as 〈*m*_*i*_〉 = *Ck*_*i*_ with $C=N/{\sum }_{j}\,{k}_{j}$ so that we satisfy ${\sum }_{i}\,\langle {m}_{i}(\tau )\rangle =N$. Topologically, the connectivity distribution determines the asymptotic state for the mean occupation number at each node 〈*m*_*i*_(*t* → ∞)〉. Therefore, for a *scale free* network we obtain a power law distribution for the asymptotic mean occupation number. However, for a directed network the analysis is not that trivial, and there seems to be no simple connection to a topological property of the network. We plan to analyze this in detail in a future manuscript.

### The Master Equation

We now construct the Master equation, that describes the evolution of the probability of occupation at each of the nodes. We start by defining the vector $\overrightarrow{{\bf{n}}}$ = [*n*_1_, *n*_2_, ..., *n*_*M*_] that represents a given occupation of the nodes of the system, which has probability *P*($\overrightarrow{{\bf{n}}}$, *t*) to occur at the iteration *t*. The convention is that 0 ≤ *n*_*i*_ ≤ *N* for all *i* = 1, ..., *M*, and satisfy the restriction

$$N=\sum _{i=1}^{M}\,{n}_{i}.$$

We also consider all vectors of the type ${\overrightarrow{{\rm{\Omega }}}}_{i,j}$ = [0, 0, ..., *n*_*i*_ = 1, .., *n*_*j*_ = −1, ...], whose components are equal to 0, except for *n*_*i*_ = +1 and *n*_*j*_ = −1. Using this definition, we can write the evolution of the probabilities as

14 $$P(\overrightarrow{{\bf{n}}},t+\mathrm{1)}=\sum _{{\overrightarrow{{\rm{\Omega }}}}_{i,j}}\,\frac{({n}_{i}+\mathrm{1)}{A}_{i,j}}{N{k}_{i}}P(\overrightarrow{{\bf{n}}}+{\overrightarrow{{\rm{\Omega }}}}_{i,j},t\mathrm{).}$$

It is easy to show that the evolution equations satisfy probability conservation

15 $$\sum _{\overrightarrow{{\bf{n}}}}\,P(\overrightarrow{{\bf{n}}},t+\mathrm{1)}=\sum _{\overrightarrow{{\bf{n}}}}\,P(\overrightarrow{{\bf{n}}},t\mathrm{).}$$

Once we know *P*($\overrightarrow{{\bf{n}}}$, *t*), we can compute the expectation value

16 $$\langle {n}_{i}(t)\rangle =\sum _{\overrightarrow{{\bf{n}}}}\,{n}_{i}P(\overrightarrow{{\bf{n}}},t),$$

the covariance matrix

17 $$\langle {\sigma }_{i,j}^{2}(t)\rangle =\sum _{\overrightarrow{{\bf{n}}}}\,({n}_{i}-\langle {n}_{i}(t)\rangle )({n}_{j}-\langle {n}_{j}(t)\rangle )P(\overrightarrow{{\bf{n}}},t)$$

and the occupation probability of a given node

18 $$P({n}_{i},t)=\sum _{[{n}_{1}\mathrm{,..}{n}_{i-1},{n}_{i+1}\mathrm{,..,}{n}_{M}]}P(\overrightarrow{{\bf{n}}},t\mathrm{).}$$

The steady state asymptotic solution can be defined as *P*_*e*_($\overrightarrow{{\bf{n}}}$) = *P*($\overrightarrow{{\bf{n}}}$, *t* + 1) = *P*($\overrightarrow{{\bf{n}}}$, *t*) for all $\overrightarrow{{\bf{n}}}$. The number of equations for a given value of *N* and *M* is

19 $${N}_{{\rm{eq}}}=\frac{1}{M!}{{\rm{\Pi }}}_{i=1}^{M}(N+i),$$

so that in general the number of equations required to find the asymptotic solution grows very quickly as *N*^*M*^, making it increasingly difficult to evolve large systems.

**Figure 2:**
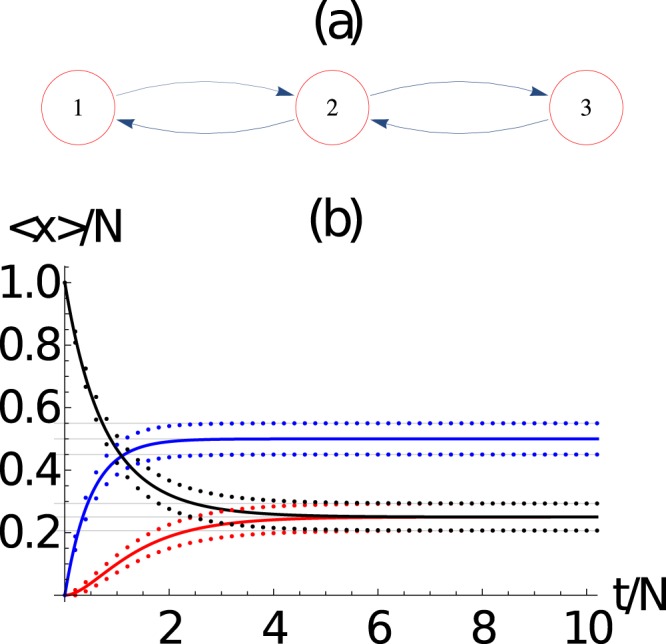
(**a**) Three-node network, in which the arrows determine the directed connectivity of the network. (**b**) Evaluation of the ensemble average number of packets at each node 〈*n*_*i*_(*t*)〉 (continuous line) and their standard deviation 〈*n*_*i*_(*t*)〉 ± *σ*_*ii*_ (dotted curves), as a function of time. The horizontal dotted lines are the corresponding values obtained from the asymptotic solution. Here we use *N* = 100 packets, which corresponds to *N*_eq_ = 1326 coupled equations. We also observe the evolution of the average number of packets at each node 〈*n*_*i*_(*t*)〉 obtained from the master equation and 〈*m*_*i*_(*t*)〉 obtained from the mean field approach. There is an excellent agreement between the master equation and the mean field approach. Here *i* = 1 (red), 2 (blue), 3 (black).

We show in Fig. 2(b) the evolution of the average number of packets at each node 〈*n*_*i*_(*t*)〉 and their standard deviation 〈*n*_*i*_(*t*)〉 ± *σ*_*ii*_, as a function of time, calculated at each time step from the evolution of the master equation. We have considered the network of Fig. 2(a) with *N* = 50 packets, which corresponds to *N*_eq_ = 1326 coupled equations. We compare the average number of packets at each node 〈*n*_*i*_(*t*)〉 obtained from the master equation and 〈*m*_*i*_(*t*)〉 obtained from the mean field approach. We observe that the equivalent values obtained from the asymptotic solution of the master equation, as we will see below, can also be obtained exactly as the *λ*_0_ = 0 eigenvector of the dynamical matrix **B**.

**Figure 3:**
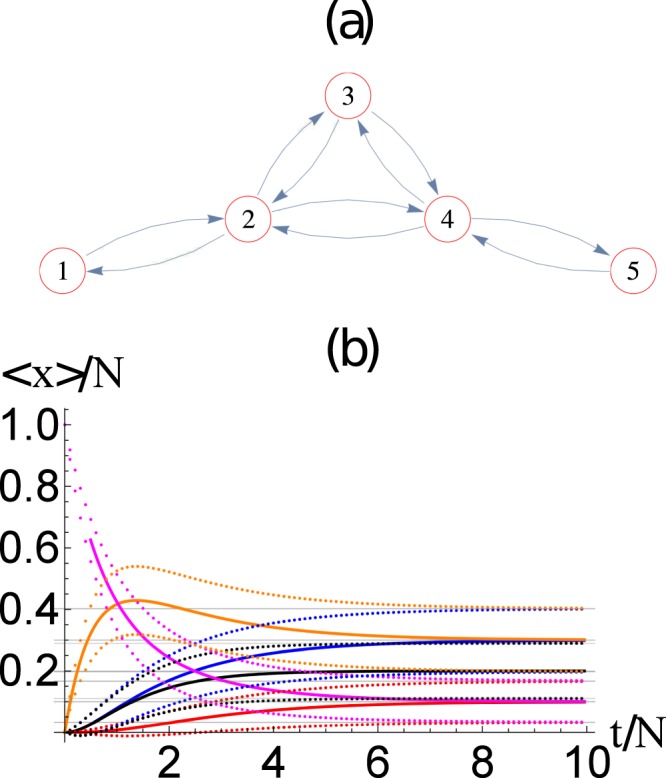
(**a**)Five-node network, in which the arrows determine the directed connectivity of the network. (**b**) Evaluation of the ensemble average number of packets at each node 〈*n*_*i*_(*t*)〉 (continuous line) and their standard deviation 〈*n*_*i*_(*t*)〉 ± *σ*_*ii*_ (dotted curves), as a function of time. The horizontal dotted lines are the corresponding values obtained from the asymptotic solution. Here we use *N* = 20 packets. The evolution of the average number of packets at each node 〈*n*_*i*_(*t*)〉 obtained from the master equation and 〈*m*_*i*_(*t*)〉 obtained from the mean field shows an excellent agreement. Here *i* = 1 (red), 2 (blue), 3 (black), 4 (orange), 5 (magenta).

We now study the network of Fig. 3(a), which displays an interesting dynamics. Figure 3(b) shows the evolution of the average number of packets at each node 〈*n*_*i*_(*t*)〉 and their standard deviation 〈*n*_*i*_(*t*)〉 ± *σ*_*ii*_, as a function of time from the evolution of the master equation. We have considered the network of Fig. 3(a) with *N* = 100 packets, which corresponds to *N*_eq_ = 10626 coupled equations. We also compare the average number of packets at each node 〈*n*_*i*_(*t*)〉 obtained from the master equation and 〈*m*_*i*_(*t*)〉 obtained from the mean field method. Again, both methods, the mean field and the master equations, provide very similar results, i.e., 〈*n*_*i*_(*t*)〉 ≈ 〈*m*_*i*_(*t*)〉.

**Figure 4:**
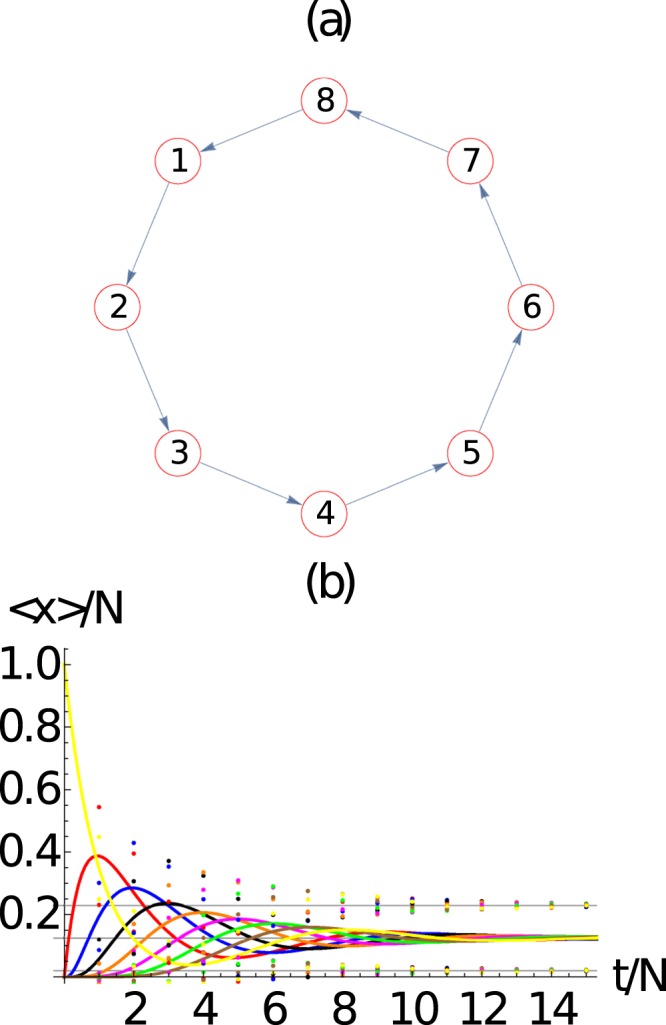
(**a**) Ring network with *M* = 8 nodes in which the arrows determine the directed connectivity of the network. (**b**) Evaluation of the ensemble average number of packets at each node 〈*n*_*i*_(*t*)〉 (continuous line) and their standard deviation 〈*n*_*i*_(*t*)〉 ± *σ*_*ii*_ (dotted curves), as a function of time. Here we use *N* = 10 packets. We observe the evolution of the average number of packets at each node 〈*n*_*i*_(*t*)〉 obtained from the master equation and 〈*m*_*i*_(*t*)〉 obtained from the mean field approach presents an excellent agreement. Here *i* = 1 (red), 2 (blue), 3 (black), 4 (orange), 5 (magenta), 6 (green), 7 (brown), 8 (yellow).

We now turn our attention to the dynamics over *small-world* networks. To construct the *small-world* networks of Watts and Strogatz^22^ we start with a ring network of *M* = 8 nodes, as shown in Fig. 4(a). The evolution of the average number of packets in the network, along with its standard deviation, is shown in Fig. 4(b), which shows an excellent agreement with the mean field approach. As expected the system converges to <*n*_*i*_> → *N*/*M* for a ring.

**Figure 5:**
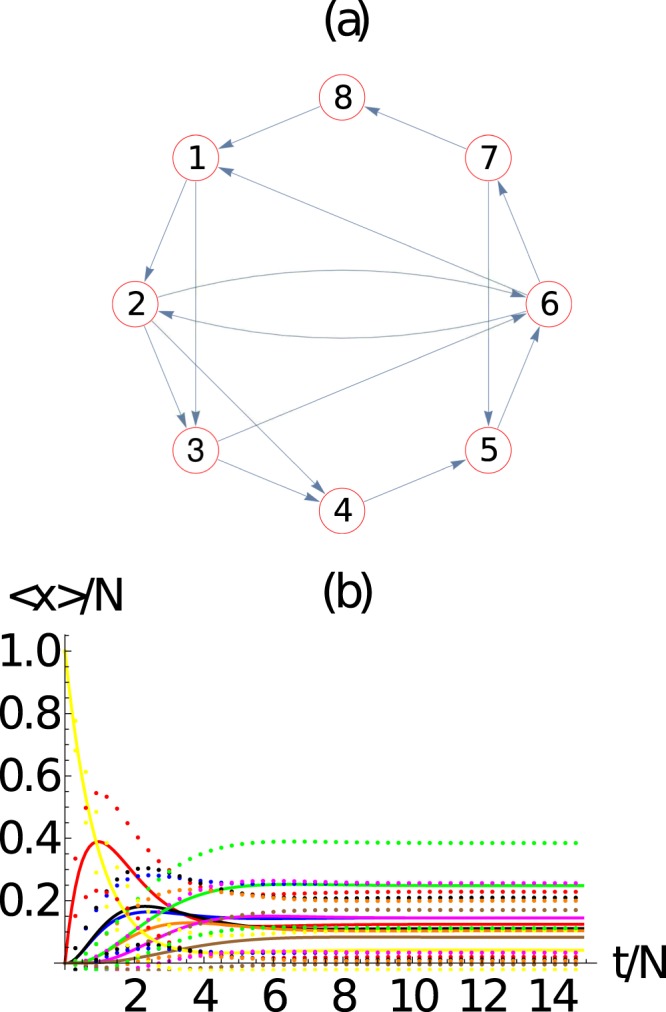
Small-world network with *M* = 8 nodes and *p* = 1, in which the arrows determine the directed connectivity of the network. (b) Evaluation of the average number of packets at each node 〈*n*_*i*_(*t*)〉 (continuous line) and their standard deviation 〈*n*_*i*_(*t*)〉 ± *σ*_*ii*_ (dotted curves), as a function of time. Here we use *N* = 10 packets. We observe the excellent agreement between the evolution of the average number of packets at each node 〈*n*_*i*_(*t*)〉 obtained from the master equation and 〈*m*_*i*_(*t*)〉 obtained from the mean field approach. Here *i* = 1 (red), 2 (blue), 3 (black), 4 (orange), 5 (magenta), 6 (green), 7 (brown), 8 (yellow).

For a *small-world* networks of Watts and Strogatz^22^, we start with the ring and then connect *M* × *p* distinct pairs of nodes, as shown in Fig. 5(a) for *p* = 1. These networks are called *small-world* networks because the average distance <*D*>/*M* between nodes decreases with *p*. The distance between nodes *i* and *j* is defined as the minimum number of steps required to reach node *j* from node *i* along the network, and considering the directed nature of the network. The evolution of the average number of packets in the network, along with its standard deviation, is shown in Fig. 5(b), which shows an excellent agreement with the mean field approach.

**Figure 6:**
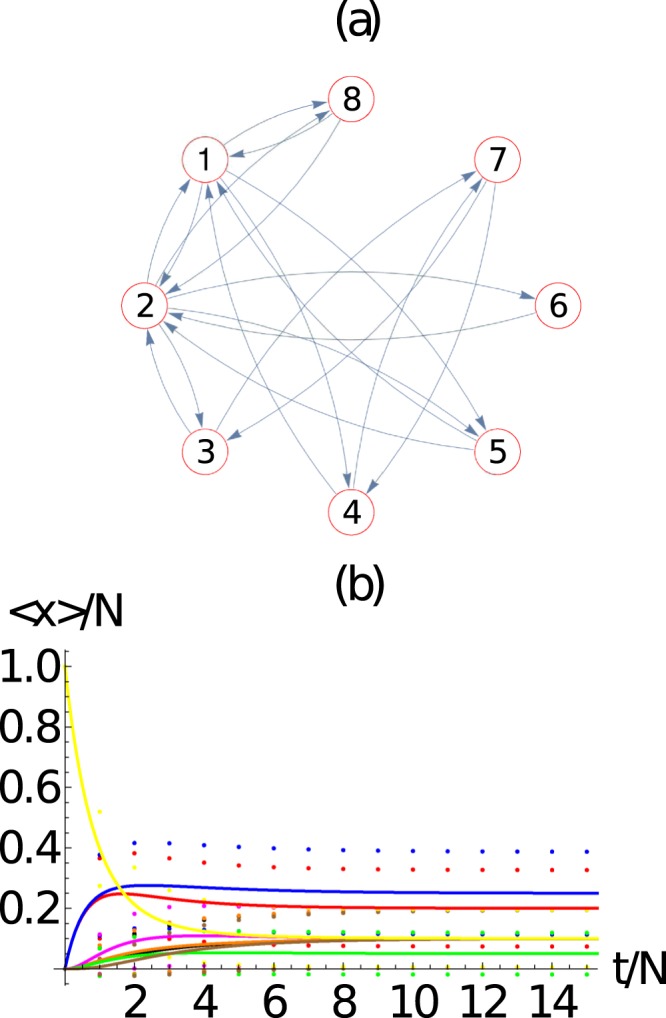
(**a**) *Erdos-Renyi* network with *M* = 8 nodes and 10 bidirectional nodes which corresponds to a density of 35% of the total possible connections (28 in total). (**b**) Evaluation of the ensemble average number of packets at each node 〈*n*_*i*_(*t*)〉 (continuous line) and their standard deviation 〈*n*_*i*_(*t*)〉 ± *σ*_*ii*_ (dotted curves), as a function of time. Here we use *N* = 10 packets. We observe that the evolution of the average number of packets at each node 〈*n*_*i*_(*t*)〉 obtained from the master equation and 〈*m*_*i*_(*t*)〉 obtained from the mean field approach exhibit an excellent agreement. Here *i* = 1 (red), 2 (blue), 3 (black), 4 (orange), 5 (magenta), 6 (green), 7 (brown), 8 (yellow).

**Figure 7:**
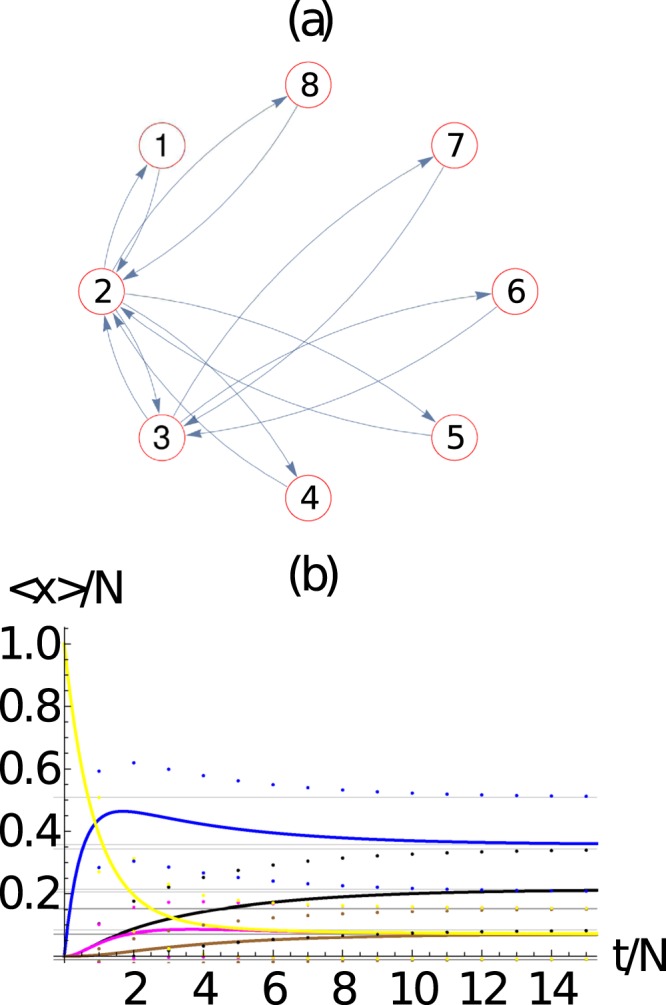
(**a**) *Scale-free* network with *M* = 8 nodes and 1 bidirectional node added at each step of the free scale network algorithm of construction with probability of attachment proportional to the vertex degree. (**b**) Evaluation of the ensemble average number of packets at each node 〈*n*_*i*_(*t*)〉 (continuous line) and their standard deviation 〈*n*_*i*_(*t*)〉 ± *σ*_*ii*_ (dotted curves), as a function of time. Here we use *N* = 10 packets. We observe the excellent agreement of the evolution of the average number of packets at each node 〈*n*_*i*_(*t*)〉 obtained from the master equation and 〈*m*_*i*_(*t*)〉 obtained from the mean field approach. Here *i* = 1 (red), 2 (blue), 3 (black), 4 (orange), 5 (magenta), 6 (green), 7 (brown), 8 (yellow).

We have studied the package evolution in two other types of networks, namely the *Erdos-Renyi* (Fig. 6) network (a complete random network) and the scale-free network (Fig. 7). Figures 6(a) and 7(a) shows the graph representation of the networks we used in our simulations. Figures 6(b) and 7(b) shows the evolution of the average number of packets at each node 〈*n*_*i*_(*t*)〉 of its respective networks. As before, there is an excellent agreement between the results obtained from the mean field approach and the master equation. It is interesting to notice that the undirected network of Fig. 5 has a larger number of different asymptotic states that the undirected networks of Figs 6 or 7. Although highly dependent on the particular connectivity distribution, it is expected that in general the breaking of the undirected symmetry of a network should produce more different asymptotic states.

It is worth noticing the overshoot phenomena that appears in Figs 3–7. We have checked that different initial conditions (i.e., varying the position in which all packages are placed at the beginning of the simulation) may modify the first part of the dynamics, producing overshoot or damping at different nodes. Hence, the overshoot that occurs at a particular node depends on the distance to the initial node, but also on the connectivity of the neighboring nodes which control how the packages are taken from each node. Of course, we checked that the asymptotic behavior is the same in all cases.

### Asymptotic equilibrium state

The equilibrium state obtained from the asymptotic solution of the master equation for the network of Fig. 2(a) is $\langle \overrightarrow{{\bf{n}}}\rangle$ = [12.5, 25, 12.5], which is the same as the one obtained from the mean field approach. The asymptotic solution for the average occupation number at each node, compared with their dynamics produced by the mean field and master equation approach is shown in Fig. 2(c), showing excellent agreements. It is interesting to notice that the corresponding covariance matrix is

$${\sigma }^{2}=[\begin{array}{rrr}9.37 & -6.26 & -3.12\\ -6.25 & 12.50 & -6.25\\ -3.12 & -6.25 & 9.37\end{array}],$$

so that the standard deviations ${\sigma }_{i}=\sqrt{{\sigma }_{ii}^{2}}$ (diagonal terms) are not all equal in the asymptotic state.

The asymptotic state obtained for the five-node network from the asymptotic solution of the master equation is $\langle \overrightarrow{{\bf{n}}}\rangle$ = [2, 6, 4, 6, 2], which is the same as the one obtained from the mean field approach. The agreement between the mean field and master equation results is excellent, as displayed in Fig. 3(c). It is interesting to notice that the corresponding covariance matrix is

$${\sigma }^{2}=[\begin{array}{rrrrr}1.8 & -0.6 & -0.4 & -0.6 & -0.2\\ -0.6 & 4.2 & -1.2 & -1.8 & -0.6\\ -0.4 & -1.2 & 3.2 & -1.2 & -0.4\\ -0.6 & -1.8 & -1.2 & 4.2 & -0.6\\ -0.2 & -0.6 & -0.4 & -0.6 & 1.8\end{array}],$$

so that the standard deviation *σ*_*i*_ (diagonal terms) are not all equal in the asymptotic state.

**Figure 8:**
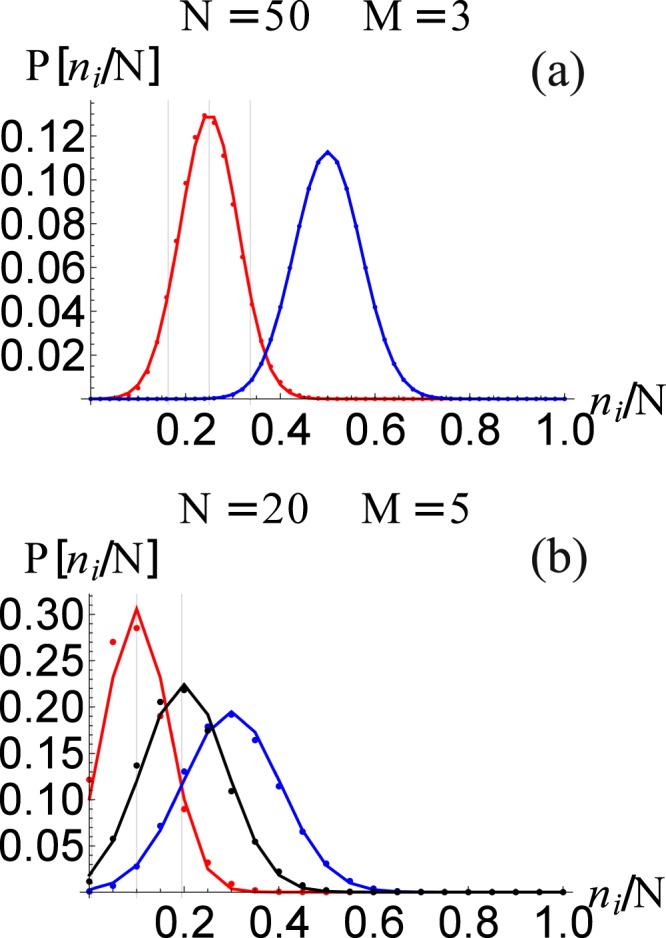
The occupation number distribution *P*(*n*_*i*_) for the (**a**) three and (**b**) five node networks for Figs 2 and 3, respectively. The dots are the values obtained from the master equation, while the continuous lines corresponds to Eq. 20 with the extrapolation from the *N* = 1 case discussed in the text. We also show 〈*n*_*i*_〉 and 〈*n*_*i*_〉 ± *σ*_*ii*_ as vertical lines.

**Figure 9:**
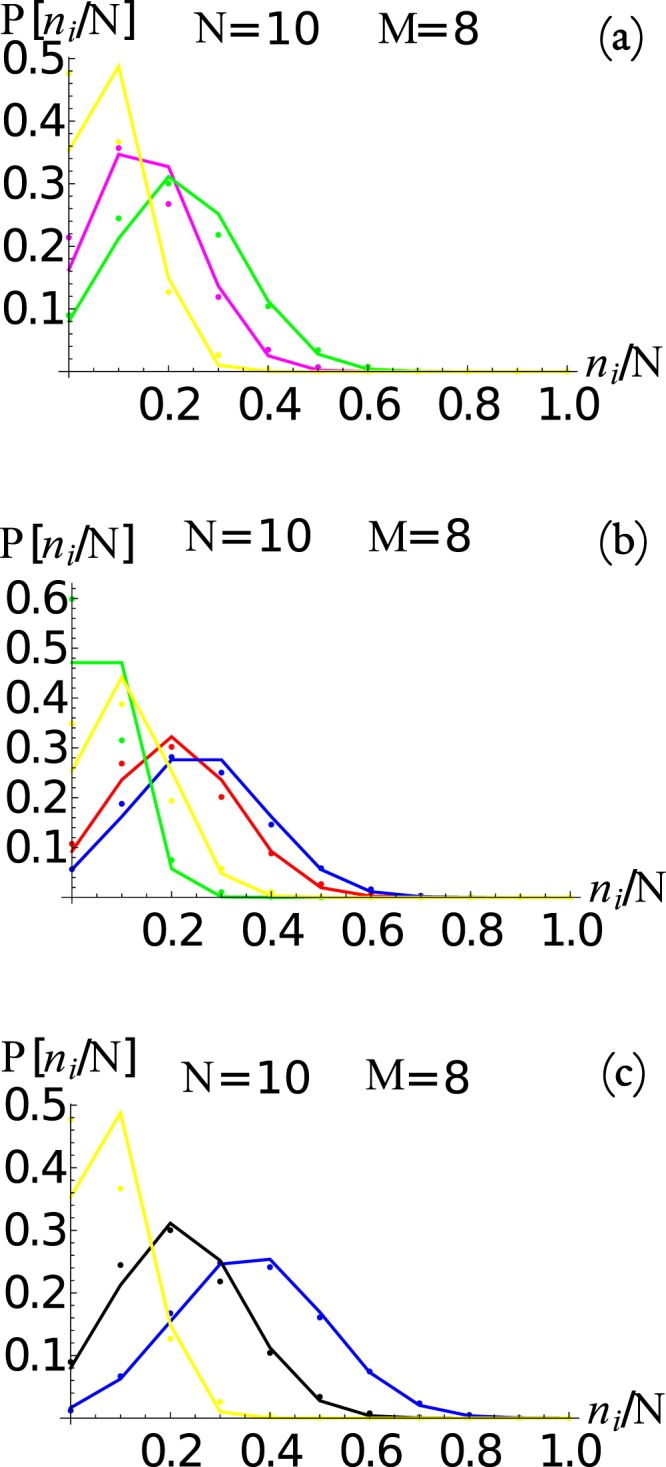
The occupation number distribution *P*(*n*_*i*_) for the (**a**) *small-world* (see Fig. 5), (**b**) *Erdos-Renyi* (see Fig. 6), and (**c**) *scale-free* (see Fig. 7) networks respectively, with eight nodes. The dots are the values obtained from the master equation, while the continuous lines corresponds to Eq. 20 with the extrapolation from the *N* = 1 case discussed in the text.

In Fig. 8(a,b), we show the asymptotic occupation number distribution *P*(*n*_*i*_) for the (a) three and (b) five node networks from Figs 2 and 3. Similarly, in Fig. 9 we show the asymptotic occupation number distribution for the (a) *small world*, (b) *Erdos-Renyi*, and (c) *scale free* networks with *M* = 8 nodes and *N* = 10 packages. Hence for small networks and packages, given the computational restrictions imposed by Eq. 19, it is reasonable to solve the master equation directly. Furthermore, for small *M* we notice that as *N* increase, the occupation number distributions at the *i*^*th*^ node approaches a normal distribution

20 $$P({n}_{i})\approx {C}_{i}\exp \,[-\frac{{({n}_{i}-\langle {n}_{i}\rangle )}^{2}}{2{\sigma }_{ii}^{2}}],$$

centered at 〈*n*_*i*_〉 with a standard deviation given by *σ*_*i*_. The normalization constant *C*_*i*_ is such that ${\sum }_{{n}_{i}\mathrm{=0}}^{N}\,P({n}_{i})=1$. The expected form of Eq. (20), for large *N*, is in agreement with Eq. (31), that is obtained from the continuous time scale description of the master equation (see Methods section for a derivation).

As *N* and *M* increase, the asymptotic state becomes increasingly more complicated to calculate, specially if we are interested in calculating the probability distribution and the standard deviation of *n*_*i*_. However, let us note that the average value of 〈*n*_*i*_〉 for the asymptotic state can be computed from the mean field approach. Hence, we can extrapolate 〈*n*_*i*_〉(*N*) as a function of *N* from the *N* = 1 case, namely

21 $$\langle {n}_{i}\rangle (N)=N\langle {n}_{i}\rangle (N=\mathrm{1).}$$

**Figure 10:**
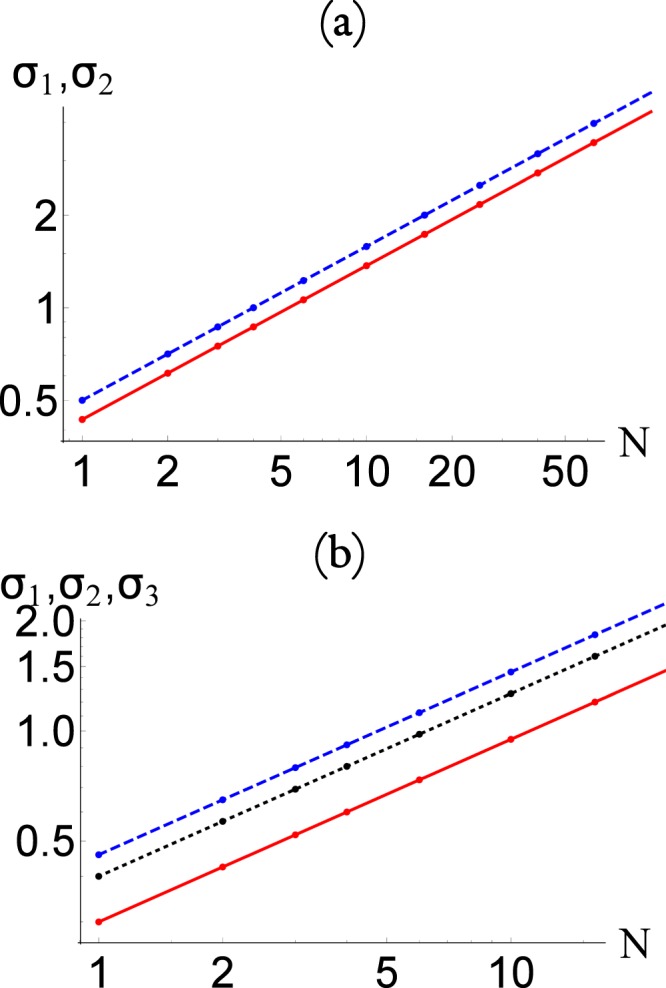
The standard deviation *σ*_*ii*_(*N*) as a function of *N* for the (**a**) three and (**b**) five node networks of Figs 2 and 3, respectively. The dots corresponds to data obtained directly from the asymptotic state calculated from the master equation, and the straight line corresponds to the re-scaling of the distribution function as discussed in the text.

Similarly, in Fig. 10(a,b), we show the standard deviations *σ*_*i*_(*N*) as a function of *N* for the (a) three and (b) five node networks for Figs 2 and 3, respectively. The continuous lines, in each case, corresponds to the scaling

22 $${\sigma }_{i,i}(N)=\sqrt{N}\,{\sigma }_{i,i}(N=\mathrm{1)},$$

which clearly gives and excellent approximation, even for relatively small values of *N*. Notice that this is expected from a stochastic system in which

23 $$\frac{{\sigma }_{ii}}{\langle {n}_{i}\rangle } \sim \frac{1}{\sqrt{N}}\mathrm{.}$$

Therefore, we see that we can compute the asymptotic state of the master equation from the *N* = 1 case and then re-scale the distribution to larger values of *N* using the scaling properties just discussed. In fact, the distribution functions displayed in Fig. 8(a,b), are constructed in this manner, showing that it is an excellent approximation, specially as *N* increases (thermodynamic limit).

The same analysis has been done for the ring and *small network* of Figs 4(c) and 5(c), respectively, which is in close agreement with Eq. 20 and the scaling from the *N* = 1 case.

Hence, if we are interested in estimating the syntactic probability distribution of the occupation number for *N* packets, it becomes of interest to solve the master equation for *N* = 1 which has *M* possible states, namely

24 $${\overrightarrow{{\bf{n}}}}_{i}=\mathrm{[0,}\,\mathrm{0,}\,\mathrm{...,}\,\mathrm{0,}\,{n}_{i}=\mathrm{1,}\,\mathrm{0,}\,\mathrm{...,}\,\mathrm{0].}$$

The evolution equation for them (using *p*_*i*_(*t*) = *P*($\overrightarrow{{\bf{n}}}$_*i*_, *t*)) is

25 $${p}_{i}(t+\mathrm{1)}=\sum _{j\ne i}^{M}\,\frac{{A}_{j,i}}{{k}_{j}}{p}_{j}(t),$$

From this equation, it becomes clear that finding the steady state *p*_*i*_(*t* + 1) = *p*_*i*_(*t*) = *p*_*i*_ for *N* = 1 is completely equivalent to finding the asymptotic mean field *λ*_0_ = 0 eigenvector of the matrix **B** given by Eq. 5, however in this case is easier to solve directly the M equations

$$\sum _{i\mathrm{=1}}^{M}\,{B}_{i,j}{p}_{j}=\mathrm{0,}$$

with the restriction ${\sum }_{i\mathrm{=1}}^{M}\,{p}_{i}=1$, than solving the complete eigensystem.

We see that there is a clear connection between the mean field approach and the master equations, which are described in the previous text. Once, we find the asymptotic states given by *p*_*i*_, we observe that *n*_*i*_ *p*_*j*_ → *δ*_*i*,*j*_ *p*_*j*_, so that the expected occupation value is

$$\langle {n}_{i}\rangle (N=\mathrm{1)}={p}_{i},$$

and the standard deviation can be found from

$$\begin{array}{rcl}{\sigma }_{i,j}^{2}(N=\mathrm{1)} & = & {p}_{i}{\delta }_{i,j}-{p}_{i}{p}_{j},\end{array}$$

which explain the negative off-diagonal values obtained above for the three and five node networks. Using these expressions, we can scale to any *N* and find 〈*n*_*i*_〉(*N*), *σ*2, and *P*(*n*_*i*_, *N*). We have to use these equations to construct the analytic approximation to the occupation probability at each node for any network, as was done for the cases of Fig. 8.

We can use this strategy, which is much less computational intensive, to re-construct the analytic approximation to the occupation probability at each node for any network, as was done in Fig. 8 for the 2 and 3 node example; and in Fig. 9 for the (a) *small world*, (b) *Erdos-Renyi*, and (c) *scale free* networks. The results show very good agreement with the master equation result, which becomes increasingly more accurate as *N* is incremented as can be observed in Fig. 8.

**Figure 11:**
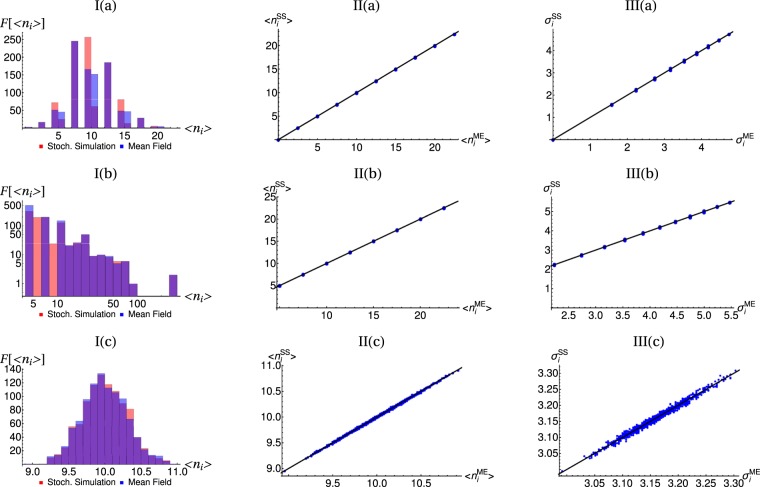
Simulations on large scale networks instances. I(a) (*small-world*), II(a) (*Erdos-Renyi*), III(a)(*scale-free*) present the probability distributions of the mean of the asymptotic states for stochastic simulation, the master equation approach, and the mean field solution. I(b), II(b), and III(b) shows the correlation between the average occupation of the master equation $\langle {n}_{i}^{ME}\rangle$ and the standard deviation of the stochastic simulation $\langle {n}_{i}^{SS}\rangle$ for its respective networks. I(c), II(c), and III(c) exhibit the correlation between the standard deviation of the master equation ${\sigma }_{i}^{ME}$ and the standard deviation of the stochastic simulation ${\sigma }_{i}^{SS}$, respectively. Simulations were run on networks of 1000 nodes (*N* = 1000) and 10^4^ packets (*M* = 10^4^). For the *small-world* case, the network was build with a re-linking probability of 0.5; for the *scale-free* case, the network was built adding 2 bidirectional node at each step of the free scale network algorithm of construction with probability of attachment proportional to the vertex degree; and the Erdos-Renyi network was build using a edge probability equal to 0.5. Sub-figures II and III were obtained studying the evolution of a particular node in network type. The *Erdos-Renyi* case was simulated with 10^5^ simulation steps, while the other two cases were simulated with 5 × 10^4^ simulation steps.

We verified our results in large scale complex networks instances of the I. *small-world*, II. *Erdos-Renyi*, and III. *scale-free* scenarios. Figure 11. summarizes these results. Panels I(a), II(a), III(a) present the frequency *F* of the average number of packets in the asymptotic states for stochastic simulation, and the mean field solution of its respective networks (*small-world*, *Erdos-Renyi*, and *scale-free*). Panels (b) show the correlation between the average occupation of the master equation $\langle {n}_{i}^{ME}\rangle$ and the standard deviation of the stochastic simulation $\langle {n}_{i}^{SS}\rangle$ for its respective networks. Panels (c) exhibit the correlation between the standard deviation of the master equation ${\sigma }_{i}^{ME}$ and the standard deviation of the stochastic simulation ${\sigma }_{i}^{SS}$, respectively. Each network contains 1000 nodes (*N* = 1000) and 10^4^ packets (*M* = 10^4^). In the *small-world* network, it was created considering a re-linking probability of 0.5; for the *scale-free* case, the network was built adding 2 bidirectional node at each step of the free scale network algorithm of construction with probability of attachment proportional to the vertex degree. The *Erdos-Renyi* network was build using a edge probability equal to 0.5. Sub-figures II and III were obtained studying the behaviors of the mean and the standard deviation of the master equation and the stochastic simulation of a particular node on the respective network. From figs. I(a), II(a), and III(a), despite small differences, the shape of the distributions are quite similar between stochastic simulation cases and mean field approach. Solid lines in sub-panels II and III are added to indicate the correlation equal to 1. To sum up, instances presented in Fig. 11 shows excellent agreement among stochastic simulation, mean field approach and master equation, allowing to observe notorious differences among different type of complex networks in large scales. It is worth noticing that the Erdos-Renyi case is simulated longer than small-world and scale-free cases: the Erdos-Renyi case was simulated with 10^5^ simulation steps, while the other two cases were simulated with 5 × 10^4^ simulation steps. The relaxation time scale $\tau =N|\Re [\tilde{<mml:mpadded xmlns:xlink="http://www.w3.org/1999/xlink" voffset="0">\lambda</mml:mpadded>}]|$ of the Erdos-Renyi network, calculated as the inverse value of the smallest nonzero eigenvalue of the B matrix, turns out to be twice as large as the time scale of the other two cases, explaining why we need to integrate longer for the ensemble averaged number of packets and its deviation to converge to the asymptotic value. This difference in time scales has to do with the fact that the degree distribution of Erdos-Renyi does not have large tails, so that the connectivity is spanned over a narrow range of values, which in turn leads to a continuos distribution of values for the ensemble averaged number of packets and its deviation. In contrast the small-world or scale-free networks has a hierarchical structure of the connectivity which is clearly evidenced by a discrete distribution for the ensemble averaged number of packets and its deviation.

## Discussion

We have generalized the Ehrenfest urn model to a complex network of urns, in which the packets or marbles move from node to node following the network connections. We have constructed the master equation for the evolution of the probability of occupation of each of the nodes in the network. The calculated occupation number at each node 〈*n*_*i*_〉 compares quite closely with analytic solution for the ensemble average evolution 〈*m*_*i*_〉 of the number of packets at each node, obtained from a mean field approach in the thermodynamic limit (namely $N\gg 1$).

We clearly observe that mean field theory provides a good approximation for the evolution of the ensemble averaged number of packets, as compared to the the evolution of the more complete master equation. However, the master equation provides a more complete description, allowing to calculate all the statistical properties of the system at any time *t*.

We also notice that the asymptotic state provided by the master equation is quite useful to find the equilibrium distribution of the occupation at each node, providing a complete statistical description at equilibrium. Furthermore, we can find scaling laws to approximate the asymptotic solution to the occupation number probability at each node from the *N* = 1 case, which involves the matrix **B** used in the mean field approach. One of the main conclusions of the manuscript is that for small networks with a small number of packets, it is necessary to find the asymptotic solution, including the correlation matrix, directly from the master equation, which is in general computationally expensive. While for large values of *N* it is possible to estimate the asymptotic state, including the correlation matrix, from the *λ*_0_ = 0 eigenvector of the matrix **B** with *N* = 1, as the whole distribution becomes normal as *N* increases with the distribution parameters satisfying the scaling relations given by Eqs 21 and 22. This approximation improves as we increase the number of packets, i.e., in the thermodynamic limit. Hence, the mean field matrix **B** can be use to estimate not only the average occupation number, but also the occupation probability distribution, and in particular the standard deviation of the average occupation number.

By comparing the mean field evolution of the network of Fig. 2 with the networks of Figs 3, 4 and 5, we observe that there is an overshoot phenomena that occurs before the system reaches the asymptotic dynamics. However, the initial condition can, and the distance from the node that contains all packets initially, also determine if there is overshoot or not. For example, if we take the network of Fig. 3, there are 3 non-equivalent nodes in which we can initially place all the packages, namely, nodes 1, 2, and 3. In this sense initially placing all the packages in node 4 is equivalent to placing them in node 2. Similarly, node 5 is equivalent to node 1. Hence, initially placing all the packages in node 1, 2, 4, and 5 produce an overshoot phenomena in nodes 2, 1, 5, and 4, respectively. However, initially placing all the packages in node 3 does not produce the overshoot phenomena. The reason for the overshoot is the following: for example, when we initially place all packages in node 1, the overshoot phenomenon occurs in node 2 as all the packages need to pass through node 2 before they can get distributed to the rest of the network. The opposite argument applies for the nonexistence of the overshoot phenomena when initially placing all packets in node 3. Hence, the topology of the network and the initial condition control the existence of this overshoot phenomenon, however, the asymptotic behavior is robust in all cases.

Finally, it is interesting to mention that the fact that the standard deviation of the occupation number at each node is not uniform in the asymptotic states, proves that the *equal a priori probabilities* proposed by Boltzmann does not apply for the transport in these networks, unless there is an underlying symmetry. The complex topology of the network provides a way to equilibrate fluctuations that become non-uniform throughout the system. This observation may have relevant implications in the understanding of the statistical mechanics of transportation networks. For example, as car change lanes in a 3 lane street, we would expect not only that the central lane will be more occupied on average than the other two lanes, but also that its fluctuations will also be larger.

## Methods

Let us assume that temporal evolution of the probability *P*($\overrightarrow{n}$, *t*) given by Eq. (14) can be written in a continuous timescale as a transition equation

26 $$\frac{d}{dt}P(\overrightarrow{n},t)=\sum _{\overrightarrow{n}\text{'}}\,({W}_{\overrightarrow{n},\overrightarrow{n}\text{'}}P(\overrightarrow{n}\text{'},t)-{W}_{\overrightarrow{n}\text{'},\overrightarrow{n}}P(\overrightarrow{n},t)),$$

where ${W}_{\overrightarrow{n},\overrightarrow{n}^{\prime} }({W}_{\overrightarrow{n}^{\prime} ,\overrightarrow{n}})$ is the transition probability of the process $\overrightarrow{n}$ → $\overrightarrow{n}^{\prime}$($\overrightarrow{n}^{\prime}$ → $\overrightarrow{n}$). Thus from

27 $$\frac{d}{dt}\sum _{\overrightarrow{n}}\,P(\overrightarrow{n},t)=\sum _{\overrightarrow{n},\overrightarrow{n}\text{'}}\,({W}_{\overrightarrow{n},\overrightarrow{n}\text{'}}P(\overrightarrow{n}^{\prime} ,t)-{W}_{\overrightarrow{n}\text{'},\overrightarrow{n}}P(\overrightarrow{n},t))=0,$$

we see that the total probability is conserved. From Eqs (16) and (26), we can write

28 $$\begin{array}{c}\frac{d}{dt}\langle {m}_{i}(t)\rangle =\sum _{\overrightarrow{n}}\,{n}_{i}\frac{dP(\overrightarrow{n},t)}{dt},\\ \,\,\,\,=\sum _{\overrightarrow{n},\overrightarrow{n}^{\prime} }\,{n}_{i}({W}_{\overrightarrow{n},\overrightarrow{n}^{\prime} }P(\overrightarrow{n}^{\prime} ,t)-{W}_{\overrightarrow{n}^{\prime} ,\overrightarrow{n}}P(\overrightarrow{n},t)).\end{array}$$

Now let us suppose that close to the steady state the transition probabilities ${W}_{\overrightarrow{n},\overrightarrow{n}^{\prime} }\to 0$ and $({W}_{\overrightarrow{n}^{\prime} ,\overrightarrow{n}})\to \omega$ (according to the ergodic theorem), thus the Eq. (28) takes the form

$$\frac{d}{dt}\langle {m}_{i}(t)\rangle =-\omega \sum _{\overrightarrow{n}\text{'}}\,{n}_{i}P(\overrightarrow{n},t)=-\,\omega \langle {m}_{i}(t)\rangle ,$$

so that the solution is 〈*m*_*i*_(*t*)〉 = *e*^−*ωt*^〈*m*_*i*_(0)〉. Comparing with Eq. (9), we note that the transition probability $\omega =|\Re [\tilde{<mml:mpadded xmlns:xlink="http://www.w3.org/1999/xlink" voffset="0">\lambda</mml:mpadded>}]|/N$. Furthermore, around the steady state $\overrightarrow{n}$, the master equation for the probability can be approximated as

29 $$\frac{d}{dt}P(\overrightarrow{n},t)=\omega \sum _{\overrightarrow{\rho }}\,(P(\overrightarrow{n}+\overrightarrow{\rho },t)-P(\overrightarrow{n},t)),$$

30 $$\begin{array}{c}\approx \,\omega ((\overrightarrow{\rho }\cdot \nabla )P(\overrightarrow{n},t)+\frac{1}{2}{(\overrightarrow{\rho }\cdot \nabla )}^{2}P(\overrightarrow{n},t))\\ =\,\frac{\omega }{2}{(\overrightarrow{\rho }\cdot \nabla )}^{2}P(\overrightarrow{n},t\mathrm{).}\end{array}$$

for small $\overrightarrow{\rho }$. For the steady state, ($\overrightarrow{\rho }$⋅∇)*P*($\overrightarrow{n}$, *t*) = 0, since at equilibrium the steady state is the most probable configuration. Hence, a direct calculation shows that the solution of the above equation is given by

31 $$P(\overrightarrow{n},t)=\frac{{P}_{0}}{\sqrt{t}}{e}^{-{(\overrightarrow{n}-\langle \overrightarrow{n}\rangle )}^{2}\mathrm{/2}\omega t},$$

which implies that the expected distribution at the steady state configuration should be a Gaussian in the thermodynamic limit.
